# Autophagy inhibition potentiates the anti-angiogenic property of multikinase inhibitor anlotinib through JAK2/STAT3/VEGFA signaling in non-small cell lung cancer cells

**DOI:** 10.1186/s13046-019-1093-3

**Published:** 2019-02-12

**Authors:** Lijun Liang, Kaiyuan Hui, Chenxi Hu, Yixuan Wen, Shikun Yang, Panrong Zhu, Lei Wang, Youyou Xia, Yun Qiao, Wen Sun, Jiayan Fei, Ting Chen, Fenghua Zhao, Baocheng Yang, Xiaodong Jiang

**Affiliations:** 1 Department of Oncology, The Affiliated Lianyungang Hospital of Xuzhou Medical University, Lianyungang, 222000 Jiangsu China; 2 Hepatobiliary/Liver Transplantation Center, the First Affiliated Hospital of Nanjing Medical University, Nanjing, 210029 China; 3 Key Laboratory on Living Donor Liver Transplantation of National Health and Family Planning Commission of China, Nanjing, 210029 China; 4 Department of Radiology 3, General Hospital of Xuzhou Coal Mining Group, the Second Affiliated Hospital of Xuzhou Medical University, Xuzhou, 221002 China; 5 Jiangsu Provincial Institute of Health Emergency, Xuzhou Medical University, Xuzhou, 221002 Jiangsu China

**Keywords:** Anlotinib, Autophagy, NSCLC, Apoptosis, Anti-angiogenesis, VEGFA

## Abstract

**Background:**

The efficacy and safety of multikinase inhibitor anlotinib have been confirmed in the treatment of advanced non-small cell lung cancer (NSCLC). However, the direct functional mechanisms of tumor lethality mediated by anlotinib were not fully elucidated, and the underlying mechanisms related to resistance remain largely elusive.

**Methods:**

Cell viability, colony formation, apoptosis and tumor growth assays were performed to examine the effect of anlotinib on lung cancer cells in vitro and in vivo. The punctate patterns of LC3-I/II were detected by confocal microscopy. HUVECs motility was detected using Transwell and scratch wound-healing assay. To visualize the microvessels, tubular formation assay was performed. The expression of LC3-I/II and beclin-1 and the changes of JAK2/STAT3/VEGFA pathway were detected by western blotting. The VEGFA levels in tumor supernatant were measured by ELISA.

**Results:**

Anlotinib treatment decreased cell viability and induced apoptosis in Calu-1 and A549 cells. Moreover, anlotinib induced human lung cancer cell autophagy in a dose- and time-dependent manner. Blocking autophagy enhanced the cytotoxicity and anti-angiogenic ability of anlotinib as evidenced by HUVECs migration, invasion, and tubular formation assay. Co-administration of anlotinib and chloroquine (CQ) further reduced VEGFA level in the tumor supernatant, compared with that of anlotinib or CQ treatment alone. When autophagy was induced by rapamycin, the JAK2/STAT3 pathway was activated and VEGFA was elevated, which was attenuated after deactivating STAT3 by S3I-201. Further in vivo studies showed that anlotinib inhibited tumor growth, induced autophagy and suppressed JAK2/STAT3/VEGFA pathway, and CQ enhanced this effect.

**Conclusion:**

Anlotinib induced apoptosis and protective autophagy in human lung cancer cell lines. Autophagy inhibition further enhanced the cytotoxic effects of anlotinib, and potentiated the anti-angiogenic property of anlotinib through JAK2/STAT3/VEGFA signaling.

## Background

Lung cancer remains the most commonly diagnosed cancer and the leading cause of cancer mortality worldwide. Based on GLOBOCAN estimates, there were about 2.1 million new lung cancer cases and 1.8 million deaths predicted in 2018 [1]. Of all the lung cancers, non-small cell lung cancer (NSCLC) accounts for 80 to 85%, and most of the patients present with locally advanced or metastatic disease at the initial diagnosis [2]. The advent of novel treatments, such as targeted therapy and immune checkpoint blockade, have transformed the management of care and significantly improved the therapeutic outcomes of patients with advanced NSCLC [3]. However, the prognosis still remained poor with a 5-year survival rate of about 19.3% [4]. Therefore, it is necessary and urgent to uncover new therapeutic strategies and drugs.

Anlotinib (AL3818) is a novel orally administered multikinase inhibitor that targets vascular endothelial growth factor receptor (VEGFR) 1 to 3, fibroblast growth factor receptor 1 to 4, platelet-derived growth factor receptors, and stem cell factor receptor (c-kit). Thus, it is considered as a broad spectrum drug with inhibitory effects on neo-angiogenesis and tumor progression [5–7]. Anlotinib has been approved in China as a third-line treatment option for patients with advanced NSCLC [8–10]. Although single agent anlotinib could improve both progression-free survival and overall survival in the phase III ALTER-0303 trial, it extended an overall survival duration of only 3 months [10]. Therefore, a more in-depth understanding regarding the underlying mechanisms of both antitumor effects and acquired resistance to anlotinib is necessary, which may in turn provide new insights to further improve the efficacy of this compound in the treatment of NSCLC.

Macroautophagy (hereafter referred to as autophagy) is an evolutionarily ancient and highly conserved catabolic process that transports cellular proteins and organelles to facilitate lysosomal degradation pathway [11]. Autophagy occurs at low basal levels in virtually all cells and is typically induced under starvation. It is initially considered to perform homeostatic functions or a survival strategy that recycles the cellular components to meet energy requirements [12]. Autophagy is usually activated in tumor cells during anticancer therapies, such as radiation, chemotherapy and target therapy [13]. This in turn mediates either autophagic cell death, probably through over-activation of self-digestion, which is considered to be Type II programmed cell death [14] or an important mechanism of drug resistance by supporting the survival of tumor cells [15]. Therefore, autophagy has opposing functions, context-dependent and stimulus-dependent roles in cancer, and both stimulation and inhibition of autophagy have been proposed as cancer therapies [13].

Accumulating evidences indicated that JAK2/STAT3 pathway are involved in tumorigenesis and angiogenesis [16, 17]. VEGF family was considered plays a crucial role in tumor angiogenesis, and VEGFA mediates the leading role [18]. Importantly, recent studies also reveal that autophagy can induce JAK2/STAT3 activation [19]. Hence, we were interested in evaluating the effects of anlotinib on autophagy status and the relationship between JAK2/STAT3 pathway activated by autophagy and anti-angiogenic capacity especially VEGFA expression. Moreover, in this study, we also examined whether targeting autophagy, a completely different process, could augment the therapeutic efficacy of anlotinib in terms of proliferation, apoptotic and angiogenesis.

## Methods

### Cell lines and reagents

Human umbilical vein endothelial cells (HUVECs) and human lung cancer cell lines including A549 and Calu-1 were purchased from American Type Culture Collection (Manassas, VA). Calu-1, A549, and HUVECs were cultured in 5A medium, RPMI-1640 medium and Endothelial Cell Medium (ECM), respectively. Medium was supplemented with 10% fetal bovine serum (FBS), 100 μ/mL penicillin and 0.1 mg/mL streptomycin. All cells were maintained in a humidified chamber at 37 °C in 5% CO_2_ atmosphere. All the experiments were conducted in the exponential phase of the cell.

Anlotinib was obtained from MedChem Express, USA and was diluted to the desired concentration in RPMI-1640 medium for in vitro experiments. Chloroquine (CQ), 3-methyl adenine (3-MA), rapamycin (RAPA), and STAT3 inhibitor (S3I-201) were purchased from Sigma-Aldrich Inc. (St Louis, MO).

### Cell viability assays and colony formation assay

The Cell Counting Kit-8 (CCK-8, Dojindo, Kumamoto, Japan) assay was used to measure the cell viability following the manufacturer’s instructions. Briefly, the cells at a density of 5 × 10^3^ cells/well were seeded in 96-well plates for 24 h before the starting of the treatment. The cells were incubated with indicated drugs or intervention for 24, 48, or 72 h at 37 °C. The mixture was then treated with CCK-8 reagent and incubated for another 0.5~3 h at 37 °C. The cell viability was determined by measuring the absorbance at 450 nm in a microplate reader. The median inhibitory concentration (IC50 value) was calculated using Prism 7.0 software (GraphPad Software). Each experiment was repeated thrice.

For colony formation assay, A549 and Calu-1 were placed into a 6- well plate at a density of 5 × 10^3^ cells/well and incubated with different concentrations of anlotinib at 37 °C for 14 days. After staining with 0.1% crystal violet for 30 min, the colonies were visualized and quantified.

### Apoptosis analysis

Cell apoptosis was determined by flow cytometry and hoechst staining. Flow cytometry assay was performed as previously described [20]. Briefly, the cells were stained with Annexin V/FITC- propidium iodide (PI) Apoptosis Detection kit (BD Biosciences, San Jose, CA, USA) and analyzed by flow cytometry. For hoechst staining, cells were fixed by 4% paraformaldehyde at room temperature for 10 min and then stained by Hoechst 33342 (Beyotime Biotechnology, Shanghai, China) at 37 °C for 5 min in a humidified dark chamber. The apoptotic tumor cells (hoechst-positive cells) were captured by a fluorescence microscopy (Nikon, Japan).

### Confocal microscopy

Cells were fixed using 4% paraformaldehyde at room temperature for 10 min. Then, cells were blocked with 0.5% bovine serum albumin (BSA) for 1 h at room temperature, incubated with microtubule-associated protein 1 light chain 3 (LC3) antibody (Sigma-Aldrich Inc., St Louis, MO) overnight at 4 °C, and then incubated with anti-rabbit IgG conjugated with Alexa Flour 555 (Beyotime Biotechnology, Shanghai, China) for 50 min at room temperature. Finally, the coverslips were treated with DAPI (Sigma-Aldrich Inc., St Louis, MO) and then imaged using a confocal microscopy (Nikon, Japan).

### Western blotting

Western blotting was performed on cultured cells and frozen tissues as previously described [20]. The following antibodies, Akt, phospho-Akt (p-Akt), mammalian target of rapamycin (mTOR), phospho- JAK2 (p-JAK2), STAT3 and phospho-STAT3 (p-STAT3) were purchased from Cell Signaling Technology (Beverly, MA, USA). Beclin-1 and LC3 antibodies were purchased from Sigma-Aldrich Inc. (St Louis, MO), phosphor- mTOR (p-mTOR) was purchased from Affinity (Cambridge, UK), and VEGFA was purchased from Abcam (USA). Antibodies against β-actin and HRP-conjugated secondary antibody were purchased from Proteintech (IL, USA).

### RNA interference

Human lung cancer cell lines A549 and Calu-1 were transfected with 200 nM of beclin-1-siRNA (Genepharma, Shanghai, China) using lipofectamine 2000 (Thermo Fisher Scientific, USA). After 24 h, cells were treated with anlotinib and then cultivated for another 24 h for further experiments. The siRNA sequences of beclin-1 were: sense: 5′-GUGGAAUGGAAUGAGAUUATT-3′, anti-sense: 5′-UAAUCUCAUUCCAUUCCACT-3′.

### Transwell assay, scratch wound healing assay, ELISA assay, and tubular formation assay

HUVECs motility (including migration and invasion) was detected using Transwell and scratch wound- healing assay. A549 and Calu-1 cells were treated with anlotinib combined with or without CQ for 24 h and then the tumor supernatant (without FBS) were collected for further experiments. Transwell assay was performed by an Invasion Chamber (Corning Incorporated, USA) following the manufacturer’s instructions. Briefly, 1.0 × 10^5^ HUVECs were seeded onto the upper chamber coated with matrigel (for invasion assay) or without matrigel (for migration assay) and tumor supernatant, while the lower chamber was seeded with ECM containing 10% FBS, and incubated for 48 h. Invasive and migratory cells on lower surface were stained with 0.1% crystal violet and counted in 5 random fields under the microscope. For scratch wound-healing assay, HUVECs were cultured with tumor supernatant (FBS free) in 6-well plates and reached to 90% confluence. A clean scratch across the center of the cell layer was generated using a sterile pipette tip. Photographs were taken after 24 h under microscope, and cell migration distance was estimated by Image J software.

To visualize the microvessels, HUVECs were cultured in tumor supernatant (without FBS) and seeded onto a 96-well plate (3 × 10^4^ cells/well) coated with 50 μl matrigel (Corning Incorporated, USA). Then, cells were incubated at 37 °C in 5% CO_2_. After 6 h of postseeding, tubules were photographed by microscopy and evaluated by Image Pro Plus software.

VEGFA concentrations in the tumor supernatants were determined using human VEGFA ELISA kits (Jianglaibio, Shanghai, China) according to the manufacturer’s instructions.

### Xenograft experiments and immunochemistry (IHC)

The BALB/c nude mice (5-weeks old) were purchased from Vital River Laboratory Animal Technology Co., Ltd. (Beijing, China). All experimental procedures and protocols were reviewed and approved by the Animal Care and Use Committee of Xuzhou Medical University. Mice were bred in specific pathogen-free conditions and a 12 h dark-light cycle. After acclimatization, 2 × 10^6^ Calu-1 cells were subcutaneously injected into the right flank of the mice. When both the tumors were palpable, the mice were randomly divided into four groups (four mice per group) and were administered with normal saline, anlotinib (6 mg/kg orally daily for consecutive 14 days), CQ (60 mg/kg intraperitoneally bid for 14 days), and anlotinib combined with CQ, respectively. Anlotinib tablets were kindly given as a gift by Chia Tai Tianqing Co., Ltd. (Lianyungang, China) and were crushed and dissolved with normal saline. Mice were killed 28 days after Calu-1 cell inoculation, and xenograft tumors were weighed and prepared for western blotting and IHC. IHC staining was conducted according to the manufacture’s protocol (Zhongshan Golden Bridge, Beijing, China). The results of IHC were determined by staining intensity and the number of positive cells. Antibodies used for IHC included anti-Ki67 (Absin, Shanghai, China), anti- vascular endothelial growth factor (VEGF) A (Abcam, USA), and CD34 (Abcam, USA).

### Statistical analysis

SPSS 16.0 software (Chicago, IL, USA) was used for statistical analyses. The data are presented as mean ± standard deviation. Comparisons among multiple groups were performed using one-way analysis of variance. The differences between control and treatment groups were analyzed using Dunnett’s multiple comparison test. Comparison between the two groups was performed by independent t test. *P* < 0.05 was considered to be statistically significant.

## Results

### Anlotinib suppressed growth and induced apoptosis of human lung cancer cells

**Fig. 1:**
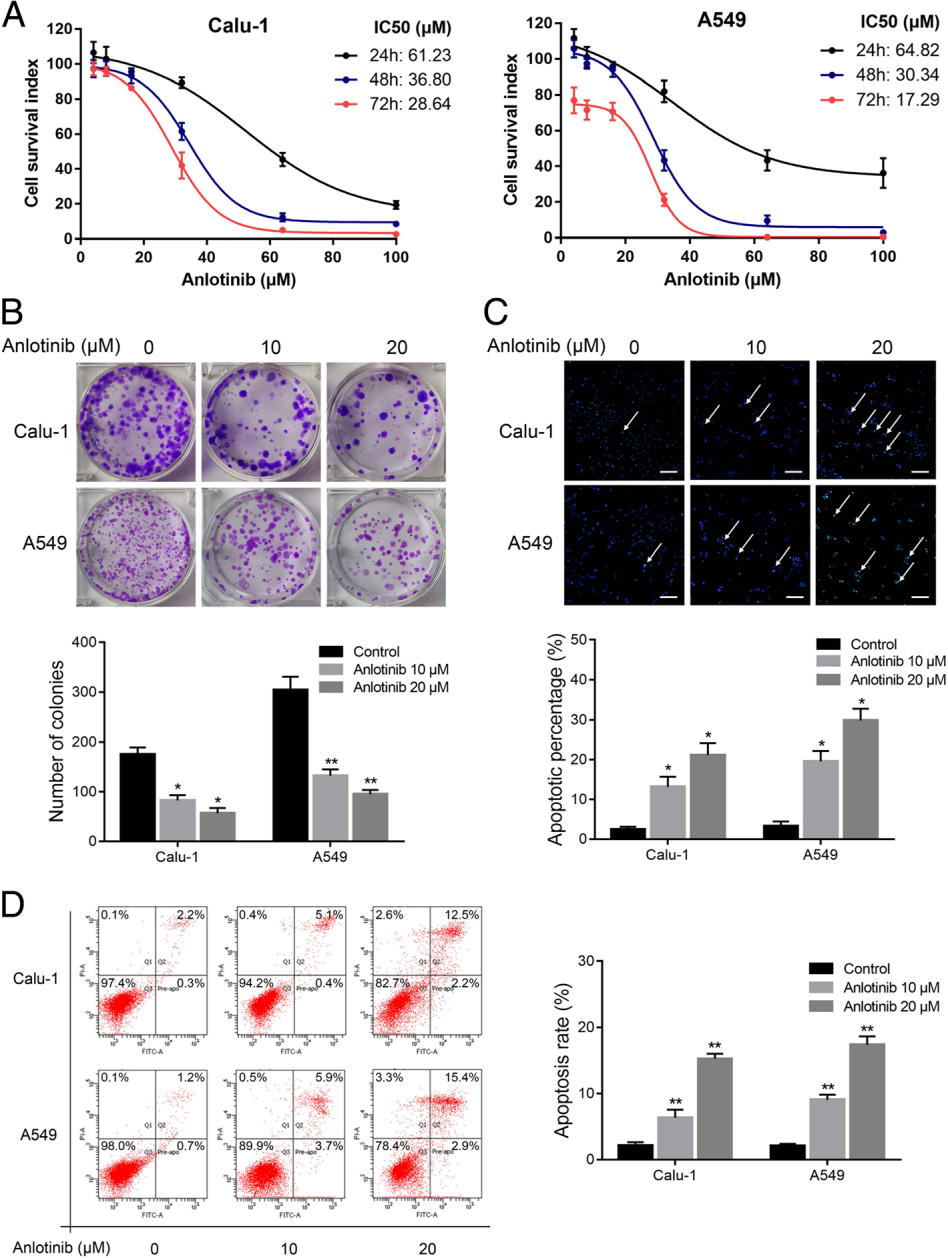
Anlotinib inhibited proliferation and induced apoptosis of human lung cancer cells. **a**, Dose-response curves of Calu-1 and A549 cells to anlotinib treatment. Cells were cultured by anlotinib at various concentrations for 24, 48 and 72 h and cell viability was detected by CCK-8 assay. **b**, Representative images of clone formation assay. **c**, Effect of anlotinib on the nuclear morphology of lung cancer cells after anlotinib (24 h) treatment was detected by Hoechst-33,258 staining. Apoptotic bodies are indicated by white arrows. **d**, The ratio of apoptotic cells was measured in Calu-1 and A549 cells after anlotinib treatment for 24 h. Apoptosis was detected by Annexin V-FITC and propidium iodide (PI) staining. The columns represent means ± SD of three independent experiments. *P < 0.05, **P < 0.01. Each experiment was performed in triplicate. Scale bar: 100 μm

To determine whether anlotinib has demonstrated direct cytotoxic effects on human lung cancer cells, CCK-8 assay was performed on Calu-1 and A549 cells. The results showed that anlotinib inhibited cell viability in a dose- and time-dependent manner and the IC50 values of the two cell lines with anlotinib for 24 h, 48 h and 72 h were shown in Fig. 1a. Colony formation was performed to further confirm the effect of anlotinib on the inhibition of lung cancer cells proliferation. The results showed fewer clone number in Calu-1 and A549 cells after anlotinib treatment (Fig. 1b).

Nuclear morphology was assessed by hoechst staining, and revealed that anlotinib induced dot-like apoptotic body formation in Calu-1 and A549 cells in a dose-dependent manner, while the nucleus of control cells was round in shape without any apoptotic bodies (Fig. 1c). As assessed by flow cytometry, anlotinib-induced apoptosis significantly when compared with the control group (Fig. 1d). Thus, these data suggested that anlotinib inhibited proliferation and promoted apoptosis of human lung cancer cells in vitro.

### Anlotinib induced human lung cancer cell autophagy

**Fig. 2:**
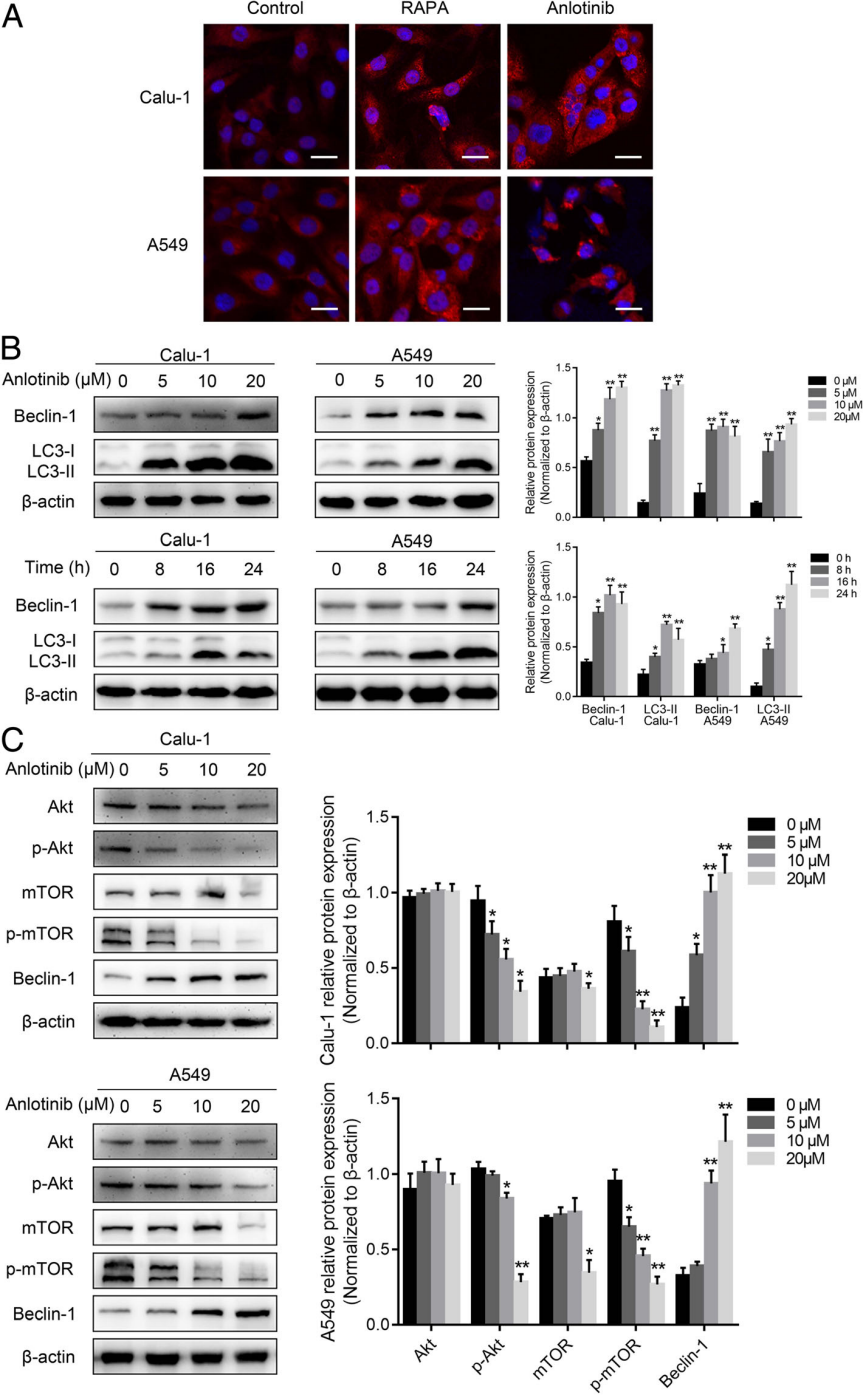
Anlotinib treatment induced autophagy in lung cancer cells. **a**, Calu-1 and A549 cells on the coverslips were treated with anlotinib or RAPA for 48 h. The punctate patterns of LC3-II were observed by confocal microscopy. **b**, Calu-1 and A549 cells were treated with anlotinib 0–20 μM for 24 h or anlotinib 20 μM for 0–24 h, and the expression levels of beclin-1 and LC3-I/II were detected by western blotting. **c**, Expression of Akt, pAkt, mTOR, p-mTOR, and beclin-1 in lung cancer cells after treatment with concentration gradient anlotinib for 24 h was detected by immunoblotting. Similar results were obtained in three independent experiments. *P < 0.05, **P < 0.01. Scale bar: 20 μm

To examine the impact of anlotinib on autophagy status in human lung cancer cells, Calu-1 and A549 cells were treated with 20 μM anlotinib and autophagy activator, RAPA, as a positive control for 24 h. LC3, a mammalian homolog of yeast atg8, is a specific protein that appears in the initial stages of autophagy, and the cytoplasmic form of LC3-I is converted to membrane-bound lipidated form LC3-II during the process of autophagy [21]. As a result, the LC3-II immunofluorescence level is regarded as a marker to find the changes in autophagosomes of the living cells. Lung cancer cells treated with anlotinib or RAPA resulted in significant increase of the dot pattern of LC3-II fluorescence compared with untreated cells (Fig. 2a). Accumulation of LC3-II and expression of beclin-1 were also detected by western blotting. As shown in Fig. 2b, both LC3-II and beclin-1 expression levels were obviously increased in a dose- and time-dependent manner. These results proved that in vitro treatment with anlotinib caused accumulation of autophagosomes in lung cancer cells.

It is widely recognized that the Akt/mTOR is a major regulatory pathway of autophagy [22]. Hence, we next examined the activity of Akt/mTOR signaling pathway in lung cancer cells. For the first time, we reported that the multikinase inhibitor anlotinib clearly blocked Akt/mTOR signaling in Calu-1 and A549 cells. After treating the concentration gradient of anlotinib for 24 h, the total expression levels of Akt proteins remained unchanged. However, high dose of anlotinib could down-regulate the expression of mTOR. In particular, the phosphorylation levels of Akt and mTOR were greatly reduced compared to the control groups in both cell lines (Fig. 2c). Concurrently, the expression of beclin-1 was increased under anlotinib treatment (Fig. 2c). In conclusion, these results demonstrated that regulation of Akt/mTOR pathway is closely related to autophagy induced by anlotinib in lung cancer cells.

### Autophagy inhibition sensitized the inhibitory effects of anlotinib in human lung cancer cells

**Fig. 3:**
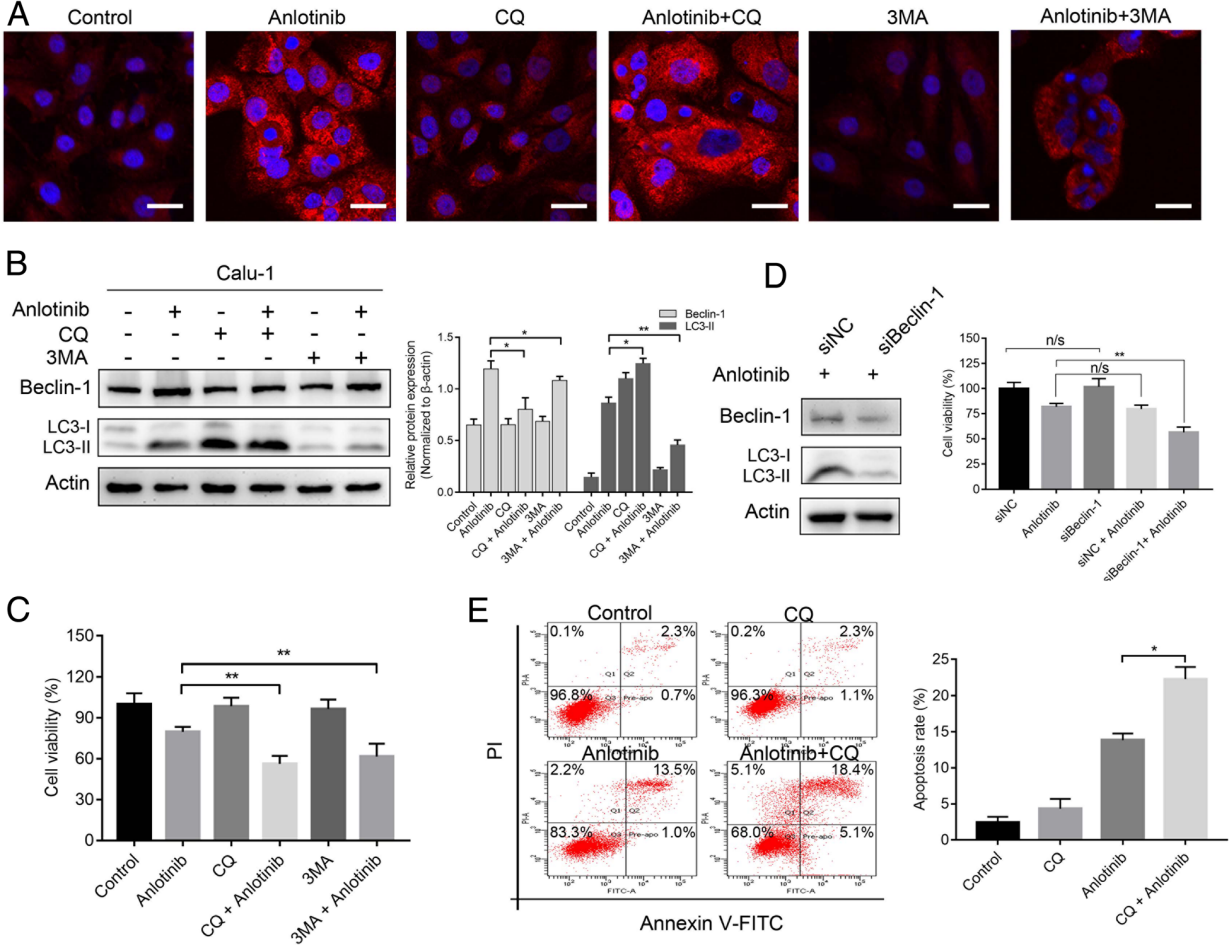
Inhibition of autophagy sensitized the inhibitory effects of anlotinib on human lung cancer cells **a**, Representative images of fluorescent LC3-II puncta as analyzed by confocal microscopy after anlotinib 20 μM treatment with or without autophagy inhibitor (CQ 25 μM and 3-MA 5 mM) for 24 h. **b**, The expressions of beclin-1 and LC3-I/II were detected using western blotting after treatment with anlotinib (20 μM) with or without 3-MA 5 mM or CQ 25 μM for 24 h. **c**, Suppression of autophagy with CQ 25 μM or 3-MA 5 mM decreased the viability of anlotinib-treated cells. **d**, The effects of cell viability after exposure to anlotinib (20 μM) with beclin-1 knockdown or siRNA negative control. **e**, Flow cytometry showed that inhibition of autophagy with CQ 25 μM or 3-MA 5 mM increased anlotinib (20 μM)-cultured cell apoptosis. Values are presented in means ± SD from three independent experiments. n/s no significant, *P < 0.05, **P < 0.01. Scale bar: 20 μm

Autophagy acts as a double-edged sword in cancer cells, i.e., it may either promote cell growth, or may induce cell death. To clarify the role of autophagy in the curative effect of anlotinib in lung cancer cell growth, two pharmacological inhibitors of autophagy were applied. The inhibitor 3-MA could inhibit the formation of autophagosome during the initial stages of autophagy process, whereas CQ could block the transition of autophagosome to autolysosome. As shown in Fig. 3a, LC3-II fluorescence punctate pattern was weakened after pretreated with 3-MA, while increased after pretreatment with CQ compared with anlotinib treatment alone. When Calu-1 cells were treated with CQ or 3-MA for 2 h and then treated with anlotinib, the expression of beclin-1 after both treatments was dramatically decreased by western blotting. However, in the 3MA pretreatment group, the cytosolic LC3-II level was reduced despite of further elevation in the CQ pretreatment group (Fig. 3b). These findings demonstrated that LC3-II accumulation induced by anlotinib resulted due to the activation of autophagosome formation, but not the inhibition of the degradation process of the autophagosome.

We next investigated the role of autophagy in the inhibitory effects of anlotinib on Calu-1 cell growth. By using CQ and 3-MA, the results showed that inhibition of autophagy obviously decreased the proliferation of anlotinib-treated cells (Fig. 3c). To confirm the cytoprotection of autophagy, we further knocked down beclin-1 expression by siRNA. As shown in Fig. 3d, Calu-1 cells transfected with beclin siRNA demonstrated decreased LC3-II expression after anlotinib conduction when compared with siRNA negative control, suggesting that beclin-1 plays a pivotal role in the autophagy of Calu-1 cells. In accordance with CQ and 3-MA, knocked down of beclin-1 enhanced the suppression ability of anlotinib in Calu-1 cells (Fig. 3d). As shown in Fig. 3e, anlotinib itself induced a modest ratio of promotion of apoptosis cells; however, in combination with CQ, the anlotinib-induced apoptosis was obviously potentiated. We explored the effect of the above intervention on A549 cells as well, and obtained similar results (data not show). In summary, these findings indicated that autophagy played a cytoprotective role, and inhibition of autophagy sensitized the inhibitory effects of anlotinib on human lung cancer cells.

### Inhibition of autophagy potentiated the anti-angiogenic properties of anlotinib

**Fig. 4:**
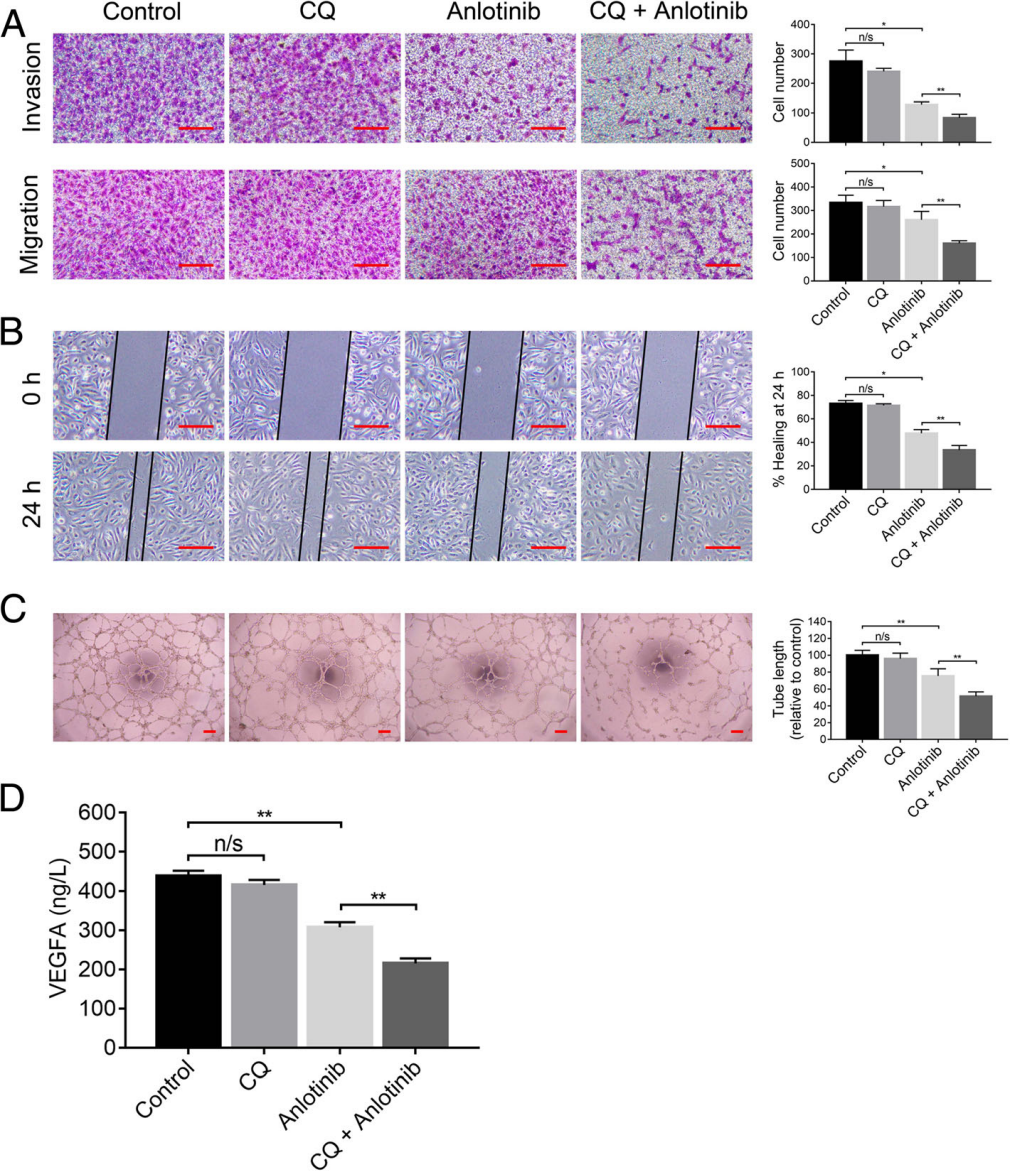
Inhibition of autophagy potentiated the anti-angiogenic properties of anlotinib. Tumor supernatant (serum free) was collected from Calu-1 cells after treatment with anlotinib and/or CQ 25 μM for 24 h. **a** to **c**, HUVECs were treated with corresponding tumor supernatant and subjected to **a** Transwell, **b**, scratch wound healing assay and **c**, tubular formation assay. **d**, The levels of VEGFA in the tumor supernatant were measured by ELISA. Data are represented as means ± SD of three independent experiments. n/s no significant, *P < 0.05, **P < 0.01. Scale bar: 100 μm

Besides direct antineoplastic effect, the effect of autophagy on the anti-angiogenic capacity of anlotinib was also evaluated. Firstly, we tested the effects of CQ on anlotinib induced HUVECs motility inhibition by Transwell and scratch wound healing assays. To culture HUVECs, tumor supernatant (serum free) was collected from Calu-1 cells after treated with anlotinib and/or CQ for 24 h. After treatment for 48 h with the corresponding supernatant, we found that Calu-1/anlotinib supernatant suppressed cell invasion and migration, while CQ further enhanced this effect (Fig. 4a). A similar tendency was observed in the wound healing ability of HUVECs (Fig. 4b).

To evaluate the effects of anlotinib combined with CQ on the sprouting of new capillaries, tubular formation assay was performed. Briefly, HUVECs were seeded on the surface of matrigel and treated with the tumor supernatant. The human umbilical endothelial tubular formation was obviously inhibited by Calu-1/anlotinib supernatant, and the inhibitory effects were further augmented when combined with CQ (Fig. 4c).

VEGF and it’s receptors were considered as the most important factors in tumor angiogenesis, and VEGFA among these mediates the leading role [18]. Therefore, we further measured VEGFA concentration in the tumor supernatant by ELISA. Co-administration of anlotinib and CQ resulted in further reduction of VEGFA level when compared with that of single anlotinib or CQ treatment (Fig. 4d). These data demonstrated that autophagy acts as a pro-angiogenic role across the anlotinib treatment process in lung cancer cells. Inhibition of autophagy could enhance the anti-angiogenesis potential of anlotinib through the reduction of VEGFA production or secretion of tumor cells.

### Autophagy preserves angiogenesis through JAK2/STAT3/VEGFA pathway

**Fig. 5:**
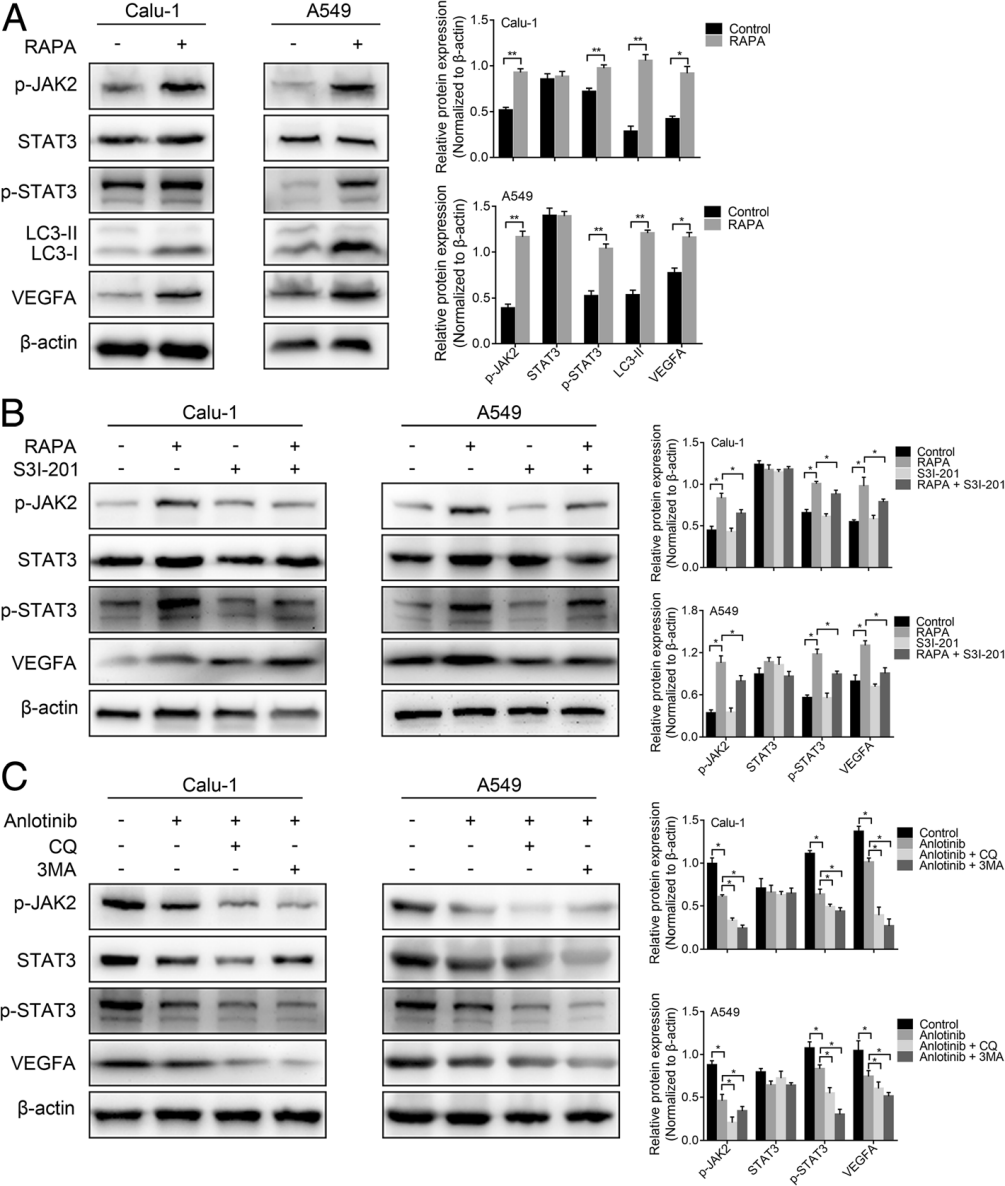
Autophagy preserved angiogenesis through JAK2/STAT3/VEGFA pathway **a**, The expressions of p-JAK2, STAT3, p-STAT3, LC3-I/II and VEGFA were detected by western blotting after the lung cancer cells were treated with 500 nM RAPA for 24 h. **b**, Western blotting showed that S3I-201,100 μM dephosphorylates STAT3 and the elevated expression of VEGFA by RAPA was reversed by S3I-201. **c**, Calu-1 and A549 cells were pre-cultured with 3-MA 5 mM or CQ 25 μM for 2 h and then incubated with 20 μM anlotinib for 24 h. The expressions of p-JAK2, STAT3, p-STAT3 and VEGFA were tested by western blotting. *P < 0.05, **P < 0.01

JAK2/STAT3 is an important signaling pathway that plays a crucial role in tumorigenesis and angiogenesis [16, 17]. Many studies have confirmed that autophagy can induce JAK2/STAT3 activation [19]; while VEGFA is a downstream target gene of JAK2/STAT3 [23, 24]. We speculated that autophagy can up-regulate VEGFA through JAK2/STAT3 pathway, thereby promoting angiogenesis. As shown in Fig. 5a, when autophagy was induced by RAPA as evidenced by increased LC3-II level, the expression of p-JAK2, p-STAT3 and VEGFA was elevated, and the total STAT3 expression was unchanged. Additionally, S3I-201, an inhibitor of STAT3, was utilized to confirm these findings. The elevation of VEGFA was attenuated after deactivating STAT3 by S3I-201 (Fig. 5b). These results suggested that autophagy may accumulate VEGFA through JAK2/STAT3 pathway activation.

Next, we investigated the role of JAK2/STAT3/VEGFA pathway in the anti-angiogenic potential of anlotinib in lung cancer cell. Lung cancer cells were treated with anlotinib or anlotinib combined with CQ or 3-MA. Figure 5c showed that anlotinib suppressed both the phosphorylation levels of JAK2 and STAT3 and the expression level of VEGFA in Calu-1 and A549 cells was also decreased after anlotinib treatment. As expected, autophagy inhibition by CQ or 3-MA further augmented the inhibition of JAK2/STAT3/VEGFA pathway by anlotinib. Taken together, these results suggested that the ability of autophagy inhibition potentiated the anti-angiogenic function of anlotinib via JAK2/STAT3/VEGFA pathway.

### Inhibition of autophagy enhanced the inhibitory effects of anlotinib on NSCLC growth in vivo

**Fig. 6:**
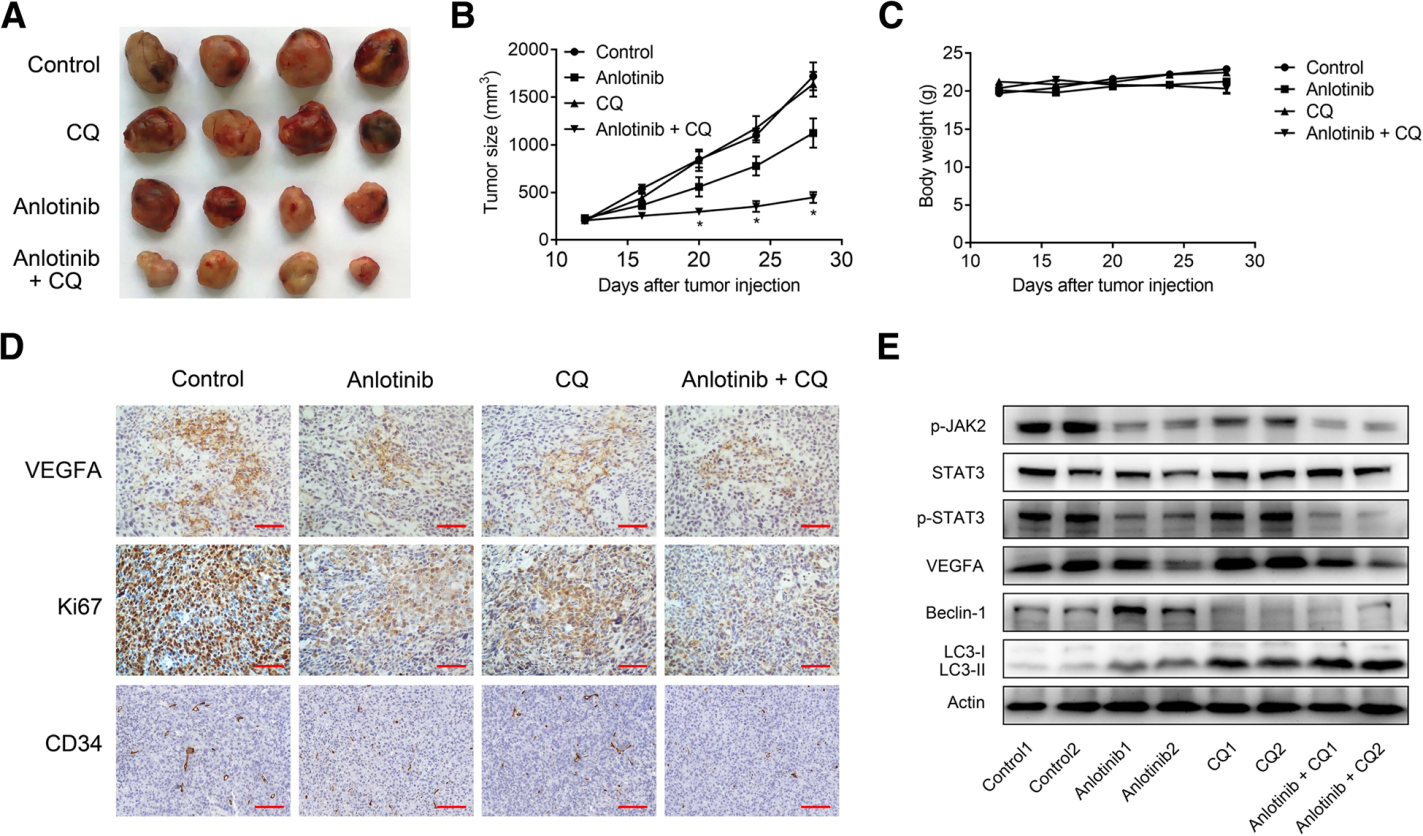
Autophagy inhibition potentiates anlotinib-induced antitumor effects in Calu-1 xenograft. **a**, The xenograft tumors were separated when the animals were killed. **b**, The tumor growth was measured every 4 days. Results were presented as means ± SD. **c**, The total body weight was monitored every 4 days. Each point represents means ± SD of body weight in each group. **d**, Representative IHC images of VEGFA, Ki67 and CD34 of the tumors. **e**, Xenograft tumor tissues from two different mice in each treatment group were tested by western blotting. These experiments were repeated thrice. *P < 0.05. Scale bar: 100 μm

To examine the therapeutic significance of autophagy inhibition for anlotinib in vivo, the mice were subcutaneously injected with Calu-1 cells to generate xenograft tumors. Co-administration of anlotinib and CQ led to superior tumor suppression when compared with administration of anlotinib alone (Fig. 6a, b). Administration of CQ alone did not slow the growth of the tumor. Besides, no significant body weight loss (Fig. 6c) or treatment-related deaths following combined therapy were observed, which demonstrated that CQ potentiated the efficacy of anlotinib in suppressing the tumor growth without additional toxicity.

Next, we investigated the autophagy and JAK2/STAT3 pathway in xenograft tumors. IHC was used to evaluate VEGFA, CD34 and proliferation marker Ki67 in tumor tissues. Anlotinib plus CQ treatments most significantly decreased the expression levels of all the three protein than other groups (Fig. 6d). JAK2/STAT3 pathway and markers of autophagy were examined by immunoblotting. The LC3-II and beclin-1 expressions were accumulated by anlotinib and CQ, indicating elevated levels of autophagy in tumor tissues. Consistent with in vitro findings, anlotinib could reduce the expression of p-JAK2, p-STAT3, and VEGFA, while CQ could dramatically enhance this effect (Fig. 6e). These results indicated that anlotinib inhibited Calu-1 xenograft growth and induced autophagy, and inhibition of autophagy potentiated the inhibitory effects of anlotinib.

## Discussion

Anlotinib is a newly developed multiple receptor tyrosine kinase (RTKs) oral small molecule inhibitor, and has become a new third-line treatment option for advanced NSCLC [9, 10]. As for the mechanism, it is proved that anlotinib can inhibit VEGF-induced migration and proliferation of HUVECs with picomolar IC50 values [5]. In this study, we demonstrated that anlotinib could suppress the viability, lead to apoptosis and induce protective autophagy in human lung cancer cell lines (Figs. 1 and 2). In addition, we demonstrated that inhibition of autophagy could further enhance the cytotoxic effects and antiangiogenic properties of anlotinib in a synergistic manner.

Currently, existing studies on anlotinib mostly focused on the antiangiogenic role and induction of tumor cell apoptosis [5, 7, 25], and data demonstrated that the effects of anlotinib on autophagy are still rare. In this study, we first discovered that anlotinib could induce autophagy in lung cancer cells in a time- and dose-dependent manner (Fig. 2a, b). Then, in further research, several pharmacological inhibitors were used including CQ and 3MA, and measure the changes of beclin-1 and LC3-II. The results showed that anlotinib not only increases the number of autophagosomes, but also activates the autophagic flux (Fig. 3a, b). It is well-known that mTOR is a central molecule that regulates autophagy [22], and its upstream inducer PI3K/Akt is the functional convergent pathway of many RTKs, which are the main targets of anlotinib [26]. Therefore, RTKs inhibitors could evoke autophagy through inhibiting PI3K/AKT/mTOR signaling activation [27] or directly targeting mTOR mRNA [28]. In our study, anlotinib could down-regulate the phosphorylation level of Akt and mTOR, high dose anlotinib could further reduce the total protein expression of mTOR, and thereby autophagy was induced in Calu-1 and A549 cells (Fig. 2c). In alignment with the present findings, Satoshi et al. [29] showed that mTOR complex 1 (mTORC1), a multisubunit complex of mTOR, was deactivated in sorafenib-mediated induction of autophagy.

A better understanding regarding the bidirectional roles of autophagy in cancer biology can help us to identify or design more powerful anti-tumor therapeutic strategies by either inducing or blocking the autophagic flux. Anlotinib treatment-elicited autophagy activation tends to have a protective role and may act as a novel mechanism related to drug resistance in human lung cancer cell lines. In this study, we revealed that autophagy inhibition using autophagy specific inhibitors (CQ and 3MA) and silencing autophagy-related gene (beclin-1) could significantly sensitize lung cancer cells to anlotinib cytotoxicity in vitro in a synergistic manner (Fig. 3c-e). Co-administration of anlotinib and CQ tended to have the most significant tumor suppressive effects when compared with anlotinib or CQ alone treatment without additional toxicity in a xenograft model (Fig. 6a-c). In accordance with our findings, several studies showed that blocking autophagy sensitized the cytotoxicity of chemotherapeutics such as cisplatin and 5-fluorouracil in some malignant tumors [30]. In contrary, other studies showed that autophagy also promoted tumor progression and resistance to treatment [31]. Although future studies are warranted to elucidate the interaction between autophagy and apoptosis in cancer cells. In the current study, we assumed that the autophagy activated by anlotinib may degrade the harmful and essential cellular proteins and organelles to suppress apoptosis and promote survival of tumor cells and this can be reversed by the administration of autophagy inhibitors.

The capacity of antitumor neo-angiogenesis is the main function of antiangiogenic drugs; however, there are rare studies that focus on the association between autophagy and the effect of inhibition of new blood vessel synthesis by multikinase inhibitors. In our study, evidenced by HUVEC migration, invasion, and tubular formation assay, anlotinib effectively inhibited angiogenesis and inhibition of autophagy by CQ and 3-MA or beclin-1 knock down enhanced the inhibitory effects of anlotinib (Fig. 4a-c), suggesting that autophagy may preserve the angiogenic potential. Consistent with our results, Abdel-Aziz et al. [32] demonstrated that the antiangiogenic response of sunitinib is augmented by co-administration of CQ via switching-off the autophagic and angiogenic machineries. Grimaldi et al. [33] showed that the synergistic effect observed when CQ combined with everolimus on endothelial cell number reduction was paralleled with increased apoptosis and reduced autophagy occurrence, but the mechanism of autophagy regulation in angiogenesis remains elusive. We discovered that co-incubation of anlotinib and CQ resulted in further reduction of VEGFA levels in tumor supernatant when compared with anlotinib or CQ treatment alone (Fig. 4d) and further in vivo experiments demonstrated similar results (Fig. 6d, e). However, in addition to the inhibition of VEGFA secretion, multiple regulation pathways may be involved in the autophagy process.

**Fig. 7:**
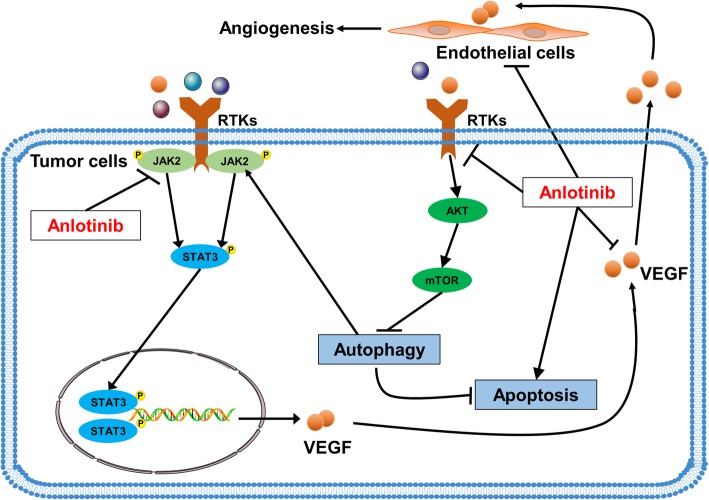
Schematic diagram of molecular mechanism of anlotinib-induced autophagy in lung cancer cells

Accumulating evidences indicated that JAK2/STAT3 are involved in tumor angiogenesis, particularly in the regulation of VEGFA. Chen et al. [34] confirmed the role of JAK2/STAT3 pathway in mediating VEGF expression upon Ginkgolide K treatment after ischemic stroke. Zhang et al. [35] demonstrated that C-X-C motif chemokine receptor 4 induced JAK2/STAT3 activation and enhanced STAT3 binding to VEGF promoter and then potentiated VEGF production in gastric cancer cells. Meanwhile, recent studies also revealed that autophagy directly regulats JAK2/STAT3 signaling pathway in lung cancer cells [19]. Besides, An et al. [36] reported that autophagy improved mesenchymal stem cells mediated vascularization in cutaneous wound healing via up-regulating paracrine VEGF. These studies assisted us to investigate the pro-angiogenic role of autophagy through JAK2/STAT3/VEGFA signaling pathway. In this study, we demonstrated that autophagy enhanced VEGFA expression of lung cancer cells via activation of JAK2/STAT3 signaling pathway both in vitro and in vivo (Figs. 5 and 7).

## Conclusion

The present study revealed for the first time that anlotinib is an effective therapeutic agent to treat lung cancer both in vitro and in vivo. Anlotinib combined with autophagy inhibition acts as an exciting and promising new therapeutic strategy. These findings provided a basis for future clinical trials to investigate on whether CQ can be used as a potential adjuvant with anlotinib in the treatment of NSCLC.

## Abbreviations

3-MA: 3-methyl adenine
BSA: Bovine serum albumin
CCK-8: Cell counting kit-8
CQ: Chloroquine
ECM: Endothelial cell medium
FBS: Fetal bovine serum
HUVECs: Human umbilical vein endothelial cells
IHC: Immunochemistry
JAK2: Janus activating kinase 2
LC3: Microtubule-associated protein 1 light chain 3
mTOR: Mammalian target of rapamycin
mTORC1: mTOR complex 1
NSCLC: Non-small cell lung cancer
PI: Propidium iodide
RAPA: Rapamycin
RTKs: Receptor tyrosine kinase
STAT3: Signal transducer and activator of transcription 3
VEGF: Vascular endothelial growth factor a
VEGFR: Vascular endothelial growth factor receptor
